# Increased Nucleotide Diversity with Transient Y Linkage in *Drosophila americana*

**DOI:** 10.1371/journal.pone.0000112

**Published:** 2006-12-27

**Authors:** Bryant F. McAllister, Amy L. Evans

**Affiliations:** Department of Biological Sciences and Roy J. Carver Center for Comparative Genomics, University of Iowa, Iowa City, Iowa, United States of America; Queens University, Canada

## Abstract

Recombination shapes nucleotide variation within genomes. Patterns are thought to arise from the local recombination landscape, influencing the degree to which neutral variation experiences hitchhiking with selected variation. This study examines DNA polymorphism along Chromosome 4 (element B) of *Drosophila americana* to identify effects of hitchhiking arising as a consequence of Y-linked transmission. A centromeric fusion between the X and 4^th^ chromosomes segregates in natural populations of *D. americana*. Frequency of the X-4 fusion exhibits a strong positive correlation with latitude, which has explicit consequences for unfused 4^th^ chromosomes. Unfused Chromosome 4 exists as a non-recombining Y chromosome or as an autosome proportional to the frequency of the X-4 fusion. Furthermore, Y linkage along the unfused 4 is disrupted as a function of the rate of recombination with the centromere. Inter-population and intra-chromosomal patterns of nucleotide diversity were assayed using six regions distributed along unfused 4^th^ chromosomes derived from populations with different frequencies of the X-4 fusion. No difference in overall level of nucleotide diversity was detected among populations, yet variation along the chromosome exhibits a distinct pattern in relation to the X-4 fusion. Sequence diversity is inflated at loci experiencing the strongest Y linkage. These findings are inconsistent with the expected reduction in nucleotide diversity resulting from hitchhiking due to background selection or selective sweeps. In contrast, excessive polymorphism is accruing in association with transient Y linkage, and furthermore, hitchhiking with sexually antagonistic alleles is potentially responsible.

## Introduction

Genome-wide surveys generally reveal a positive relationship between the local rate of meiotic recombination and the level of nucleotide diversity [1], [2], [3]. Local recombination rates determine the interdependence of neutral, beneficial and deleterious mutations, thus a reduction in recombination is accompanied by a diminished capacity for natural selection to act individually on each new mutation [4], [5], [6]. Hitchhiking of neutral variants as a consequence of selection on nearby mutations is a likely cause of spatial patterns of nucleotide diversity throughout genomes [7], [8], [9]. Reduced nucleotide variation associated with low or no recombination can extend throughout entire chromosomes, such as the “dot” chromosome in species of Drosophila [10], [11], [12], and the Y or W sex chromosome in many species [13], [14], [15], [16]. Although examples of reduced nucleotide variation in regions experiencing chronically low recombination are prevalent, there is little agreement on the impact of beneficial versus deleterious mutation and the magnitude of the selective forces responsible for structuring variation in these regions.

**Figure 1:**
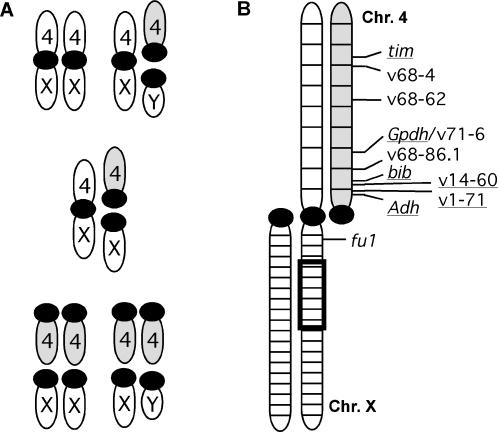
Characteristics of Chromosome 4 in *Drosophila americana.* The unfused arrangement of Chromosome 4 is highlighted in gray. (A) Karyotypic variation involving the X and 4^th^ chromosome as a consequence of the X-4 fusion. Frequency of the X-4 fusion determines the relative prevalence of each karyotype. Female karyotypes are expected to occur according to Hardy-Weinberg equilibrium based on a single locus with two alleles, and male karyotypes occur in proportion to the frequencies of the two arrangements of the X. (B) Idealized arrangement of Chromosome 4 during meiosis in a heterokaryotypic female having both X chromosome arrangements. Positions of the loci used in the analyses are indicated relative to the euchromatic subdivisions of Chromosome 4. Sequenced regions are underlined. An inversion of the X chromosome (*In(X)c*) discriminates the two lines used in the linkage analysis, and a box demarcates its approximate position.

Newly arisen sex chromosomes provide a unique system for examining patterns of sequence diversity in response to a change in the rate of recombination. After they emerge from a homologous pair of autosomes, mechanisms usually arise to suppress meiotic crossing over between X and Y chromosomes [17]. This crossover suppression creates an asymmetry in recombination rate between the X chromosome, which can recombine in females, and the Y chromosome, which is genetically isolated in males. An example of an emerging Y chromosome is present in *Drosophila americana*. Chromosome 4 (Muller element B) exists in a dynamic state shifting from an autosomal pair to a sex-chromosome pair along a latitudinal gradient of this species' range. This unique situation arises due to the presence of a derived centromeric fusion of the X and 4^th^ chromosomes that is positively correlated with latitude [18]. Unfused Chromosome 4 segregates autosomally in the absence of the X-4 fusion. An unfused 4^th^ chromosome exists as a secondary Y chromosome in males with the X-4 fusion (Fig. 1). Given the absence of crossing over in male Drosophila [19], the rate of recombination among loci on these neo-Y chromosomes is, therefore, directly influenced by the frequency of the X-4 fusion.

Reduction in nucleotide diversity is a predicted outcome of restricting unfused 4^th^ chromosomes in males as neo-Y chromosomes. In addition to the reduction expected as a consequence of a smaller effective population size, which would accrue slowly through genetic drift [20], reduction in diversity may also be driven by background selection as a result of negative selection pressure [21], by selective sweeps as a result of positive selection pressure [6], or by a combination of these and other related processes [22]. Previous comparison of nucleotide diversity between unfused and fused 4^th^ chromosomes of *D. americana* from a single northern population having a high (∼98%) frequency of the X-4 fusion revealed an apparent deficit of nucleotide polymorphism on the unfused arrangement, which was interpreted as a loss of diversity [23]. However, subsequent discovery of non-neutral patterns of sequence variation on the fused 4^th^ chromosome raises uncertainty about the assumptions underlying this contrast, because nucleotide diversity on the fused 4^th^ chromosome has been influenced by a segregating chromosomal inversion [24]. In this study, an alternative approach is used to examine the initial effects of reduced recombination on this neo-Y chromosome. Patterns of sequence diversity are contrasted among unfused 4^th^ chromosomes obtained from populations that differ in the frequency of the X-4 fusion and thus represent different degrees of chromosome-wide reductions in recombination.

The unfused arrangement of Chromosome 4 is not genetically isolated from its fused homolog. These arrangements coexist over a wide geographic region [18], and the occurrence of heterokaryotypic females with both arrangements is consistent with Hardy-Weinberg expectations [25, McAllister et al. unpublished data]. Heterokaryotypic females create ample opportunities for meiotic exchange between alternatively arranged chromosomes, so location on Chromosome 4 influences linkage relationships with the two centromeric arrangements and ultimately with the sex chromosomes (Fig. 1). Exchange between arrangements in populations with a high frequency of fused X chromosomes creates a gradient of sex linkage along Chromosome 4. Reduction in diversity on the unfused arrangement through drift, background selection or selective sweeps should be greatest in proximal chromosomal regions within northern populations. Other selective forces may generate alternative effects on sequence diversity. For example, sexually antagonistic alleles that increase fitness in males and decrease fitness in females are prone to accumulate as Y-linked polymorphisms [26]. If a sexually antagonistic allele increases to intermediate frequency by selection, but is impeded from reaching fixation, a balanced polymorphism between the antagonistic and neutral allele would be maintained at the locus. Balanced polymorphisms inflate levels of linked neutral variation [27], [28], [29]. Alternative selective forces may, therefore, be distinguishable by examining the pattern of sequence diversity along the unfused arrangement of Chromosome 4.

This study leverages the robust cline in the frequency of the X-4 fusion to examine patterns of sequence diversity shaped by recombination and Y linkage at two levels: 1) across populations with different frequencies of the X-4 fusion, and 2) along the chromosome where loci experience different levels of Y linkage. Six gene regions distributed along Chromosome 4 were assessed for patterns of sequence variation. Sequences were obtained specifically from the unfused 4^th^ chromosome in four different samples of *D. americana* from contiguous populations representing a wide spectrum in the frequency of the X-4 fusion. An overall difference in levels of sequence diversity is not evident among populations. However, a distinct pattern along the chromosome is revealed in relation to the X-4 fusion. Loci experiencing the strongest Y linkage exhibit inflated levels of sequence diversity, which is contrary to expectations from background selection and selective sweeps. Simulations demonstrate the propensity for sexually antagonistic alleles to accumulate as polymorphisms in tightly Y-linked regions of Chromosome 4. The observed pattern of sequence diversity is consistent with this form of intra-locus sexual conflict generating pervasive effects along this transient Y chromosome.

## Results

### Variation Across Populations

Contrasting populations of *D. americana* that differ in the frequency of the X-4 fusion identifies immediate effects associated with a localized reduction in the rate of meiotic recombination. Samples of the unfused 4^th^ chromosome were obtained from flies collected near rivers in the eastern sections of the contiguous states of Arkansas (FP), Missouri (HI), and Iowa (IR & SB) in the USA. This fly species inhabits riparian areas, and given that each sample site is near a tributary of the Mississippi River, the opportunity exists for a high rate of gene flow among populations. Even with this potential for gene flow, observed frequencies of the X-4 fusion exhibit large differences among these samples and are representative of more extensive sampling from the Mississippi River Valley (Table 1). Estimates of the frequencies of the X-4 fusion are 16% in the southern population represented by FP, 85% in the northerly population represented by HI, and 97% in the northernmost population represented by both IR and SB. Based on these frequencies and assuming Hardy-Weinberg equilibrium, 97% of males in the northernmost population exhibit sex linkage of Chromosome 4, whereas about 6% of females are heterokaryotypes. Sex linkage in HI and FP is almost exactly opposite (85% of males in HI and 16% of males in FP), whereas the frequency of heterokaryotypic females is about 25% in each. Variability in the frequency of the X-4 fusion creates explicit dynamics with potential consequences on patterns of sequence diversity across these populations and along Chromosome 4.

**Table 1:**
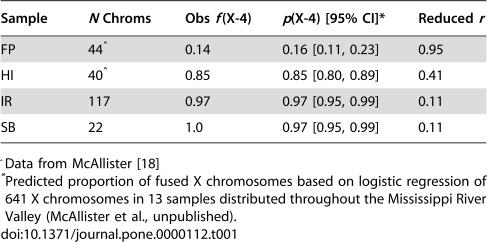
Frequency of the X-4 fusion and consequent reduction in meiotic recombination on unfused Chromosome 4.

Sample	*N* Chroms	Obs *f*(X-4)	*p*(X-4) [95% CI]*	Reduced *r*
FP	44 ^	0.14	0.16 [0.11, 0.23]	0.95
HI	40 ^	0.85	0.85 [0.80, 0.89]	0.41
IR	117	0.97	0.97 [0.95, 0.99]	0.11
SB	22	1.0	0.97 [0.95, 0.99]	0.11

^ Data from McAllister [18]

* Predicted proportion of fused X chromosomes based on logistic regression of 641 X chromosomes in 13 samples distributed throughout the Mississippi River Valley (McAllister et al., unpublished).

A high frequency of fused X chromosomes decreases the overall rate of recombination on the unfused arrangement of Chromosome 4. This effect arises from restricting the unfused 4 in males, which lack meiotic crossing over. Based on the proportion of fused X chromosomes in the populations studied here, meiotic recombination between any two loci on unfused Chromosome 4 relative to their autosomal rate is 0.95 in FP, 0.41 in HI, and 0.11 in IR/SB (Table 1), thus comparisons of FP versus IR or SB represent about a 10-fold reduction in the recombination rate per generation between any two loci on this chromosome. One potential consequence of this reduction is that the chromosomal region of reduced nucleotide diversity associated with a geographically localized selective sweep limited to unfused Chromosome 4 is expected to be about 10 times wider in the northernmost population than in the southern population [7]. An overall reduction in recombination intensifies background selection, but the degree to which its effects arise depends on the ratio of the deleterious mutation and local recombination rates [8]. Equilibrium levels of nucleotide diversity are not expected to exhibit differences among these populations if this ratio is less than 0.01 in the southern population. On the other hand, a higher rate of deleterious mutation or a lower rate of recombination is expected to generate a difference in levels of nucleotide diversity at mutation/selection equilibrium (*e.g.*, a ratio of 0.1 in FP is expected to generate over a two-fold reduction in standing level of diversity in IR/SB). Sequence diversity was compared across these populations to detect evidence of selective sweeps or background selection.

**Figure 2:**
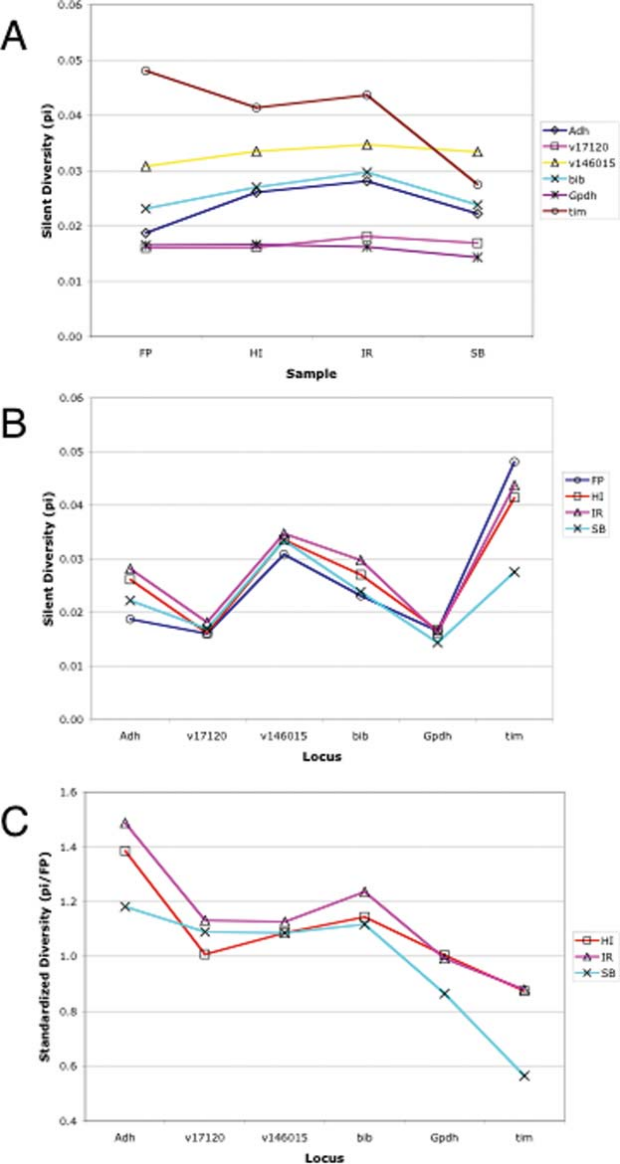
Nucleotide diversity on unfused Chromosome 4. (A) Average pairwise silent diversity across populations. Samples are arranged with FP, which has the lowest frequency of the X-4 fusion, on the left and SB, which has the highest observed frequency of the X-4 fusion, on the right. No significant heterogeneity is evident among populations. (B) Pattern of sequence diversity along Chromosome 4. Loci are arranged with the centromere to the left and telomere to the right. Average pairwise silent diversity for each sample is plotted to show heterogeneity among loci and the lack of an overall pattern relative to the centromere. (C) Standardized diversity in northern populations. Total pairwise diversity in each northern sample relative to FP is plotted for each locus. A significant relationship between chromosomal position and relative diversity is indicated by the Spearman rank correlation coefficient (*r_s_* = −0.78, 95% CI: −0.94, −0.40).

Ten unfused 4^th^ chromosomes from each sample were assayed for sequence polymorphism at six gene regions distributed along the chromosome. Descriptive statistics of nucleotide polymorphism are presented in Table 2 (sequences deposited in GenBank under accessions EF136929-EF137168). Selective sweeps and background selection are both expected to decrease sequence diversity in relation to decreased recombination. Measures of silent pairwise diversity for each gene region in each sample are presented in Figure 2A. No difference in the rank score of diversity is evident among the populations controlling for differences among genes (*p*>0.1, Friedman ANOVA). Furthermore, there is no statistical evidence (*p*>0.1, Spearman rank correlation) for a correlation between sequence diversity and relative recombination rate. Similar results with the same conclusions are obtained in analyses of segregating mutations. Shifts in the frequency spectra of segregating sites are not evident among the populations. Tajima's *D* [30] statistic is reported in Table 2, but similar results were observed for Fu and Li's *D* [31] and Fay and Wu's *H* [32]. These data indicate that strongly selected mutations, either beneficial or deleterious, have not uniformly reduced levels of nucleotide diversity or altered the distribution of segregating polymorphisms on the unfused 4^th^ chromosome in populations where it experiences reduced levels of meiotic exchange.

**Table 2:**
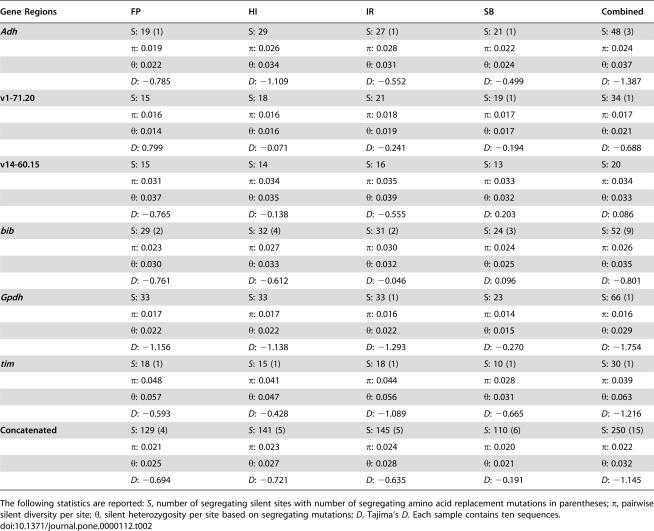
Summary statistics describing sequence diversity in four samples of *D. americana*.

Gene Regions		FP		HI		IR		SB		Combined	
***Adh***		S: 19 (1)		S: 29		S: 27 (1)		S: 21 (1)		S: 48 (3)	
		*π*: 0.019		*π*: 0.026		*π*: 0.028		*π*: 0.022		*π*: 0.024	
		*θ*: 0.022		*θ*: 0.034		*θ*: 0.031		*θ*: 0.024		*θ*: 0.037	
		*D*: −0.785		*D*: −1.109		*D*: −0.552		*D*: −0.499		*D*: −1.387	
**v1-71.20**		S: 15		S: 18		S: 21		S: 19 (1)		S: 34 (1)	
		*π*: 0.016		*π*: 0.016		*π*: 0.018		*π*: 0.017		*π*: 0.017	
		*θ*: 0.014		*θ*: 0.016		*θ*: 0.019		*θ*: 0.017		*θ*: 0.021	
		*D*: 0.799		*D*: −0.071		*D*: −0.241		*D*: −0.194		*D*: −0.688	
**v14-60.15**		S: 15		S: 14		S: 16		S: 13		S: 20	
		*π*: 0.031		*π*: 0.034		*π*: 0.035		*π*: 0.033		*π*: 0.034	
		*θ*: 0.037		*θ*: 0.035		*θ*: 0.039		*θ*: 0.032		*θ*: 0.033	
		*D*: −0.765		*D*: −0.138		*D*: −0.555		*D*: 0.203		*D*: 0.086	
***bib***		S: 29 (2)		S: 32 (4)		S: 31 (2)		S: 24 (3)		S: 52 (9)	
		*π*: 0.023		*π*: 0.027		*π*: 0.030		*π*: 0.024		*π*: 0.026	
		*θ*: 0.030		*θ*: 0.033		*θ*: 0.032		*θ*: 0.025		*θ*: 0.035	
		*D*: −0.761		*D*: −0.612		*D*: −0.046		*D*: 0.096		*D*: −0.801	
***Gpdh***		S: 33		S: 33		S: 33 (1)		S: 23		S: 66 (1)	
		*π*: 0.017		*π*: 0.017		*π*: 0.016		*π*: 0.014		*π*: 0.016	
		*θ*: 0.022		*θ*: 0.022		*θ*: 0.022		*θ*: 0.015		*θ*: 0.029	
		*D*: −1.156		*D*: −1.138		*D*: −1.293		*D*: −0.270		*D*: −1.754	
***tim***	*S*: 18 (1)		*S*: 15 (1)		S: 18 (1)		*S*: 10 (1)		S: 30 (1)		
	*π*: 0.048		*π*: 0.041		*π*: 0.044		*π*: 0.028		*π*: 0.039		
	*θ*: 0.057		*θ*: 0.047		*θ*: 0.056		*θ*: 0.031		*θ*: 0.063		
	*D*: −0.593		*D*: −0.428		*D*: −1.089		*D*: −0.665		*D*: −1.216		
**Concatenated**	*S*: 129 (4)		*S*: 141 (5)		S: 145 (5)		*S*: 110 (6)		S: 250 (15)		
	*π*: 0.021		*π*: 0.023		*π*: 0.024		*π*: 0.020		*π*: 0.022		
	*θ*: 0.025		*θ*: 0.027		*θ*: 0.028		*θ*: 0.021		*θ*: 0.032		
	*D*: −0.694		*D*: −0.721		*D*: −0.635		*D*: −0.191		*D*: −1.145		

The following statistics are reported: *S*, number of segregating silent sites with number of segregating amino acid replacement mutations in parentheses; *π*, pairwise silent diversity per site; *θ*, silent heterozygosity per site based on segregating mutations; *D*, Tajima's *D*. Each sample contains ten sequences.

### Gene Flow

One criterion for selecting the samples examined here was their potential for maintaining historically high levels of reciprocal gene flow. Consistent with this expectation, overall differentiation measured by *K_st_* is 0.001, which permutation tests reveal is not significantly different from zero (other measures of differentiation yield the same result). Pairwise comparisons among samples revealed no cases of statistically significant differentiation. The highest levels of differentiation are observed for comparisons with SB (Table 3), which is likely due to the inflation of the *K_st_* statistic by the generally low overall level of sequence diversity within this sample [33]. Specific comparisons of south (FP) versus north (HI, IR and SB combined) and among northern samples (HI, IR, SB) also exhibit no evidence of subdivision (Table 3). Individual loci also show no evidence of statistically significant differentiation (data not shown). Although these samples show a high level of differentiation for the X-4 fusion arrangement itself (*F_st_* = 0.64), these analyses did not reveal differentiation in the nucleotide variants on the unfused arrangement of Chromosome 4.

**Table 3:**
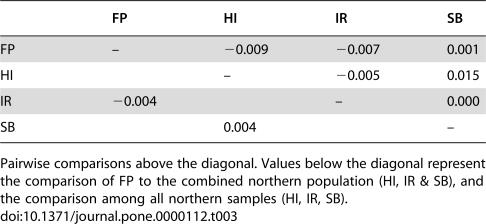
Sequence differentiation measured by *K_st_*.

	FP	HI	IR	SB
FP	–	−0.009	−0.007	0.001
HI		–	−0.005	0.015
IR	−0.004		–	0.000
SB		0.004		–

Pairwise comparisons above the diagonal. Values below the diagonal represent the comparison of FP to the combined northern population (HI, IR & SB), and the comparison among all northern samples (HI, IR, SB).

### Variation Among Loci

Patterns of sequence diversity along the unfused arrangement of Chromosome 4 were examined using the six sequenced gene regions. Silent diversity is significantly heterogeneous among the sequenced loci (*p*<0.01, Freidman ANOVA). However, there is no detectable relationship between sequence diversity and position relative to the centromere (*p*>0.1, Spearman rank correlation, Fig. 2B). Several factors are potentially responsible for the observed differences in segregating nucleotide variation among gene regions (*e.g.*, mutation rate, local recombination rate, and codon usage). Dividing by divergence estimates is one method of standardizing diversity measures; however, the ratio of polymorphism to divergence (using *D. virilis* as an outgroup) exhibits a similar pattern of heterogeneity among loci (Supporting Figure S1).

Relative measures contrasting populations for variance at microsatellite loci have been utilized effectively to identify loci exhibiting population-specific reductions in variance associated with selective sweeps [34]. Comparing across loci using a relative measure corrects for locus-specific effects, such as a different mutation rate [35]. A similar approach was applied to these sequence data using FP to standardize sequence diversity in the northern samples, since FP was selected *a priori* to represent a population where the low frequency X-4 fusion is not expected to influence sequence diversity on what is essentially an autosome. Using FP to obtain relative measures of sequence diversity for HI, IR and SB, a clear pattern emerges where sequence diversity in northern samples is elevated relative to FP for proximal loci and is equal or lower than FP for distal loci (Fig. 2C). The Spearman rank correlation between standardized diversity and position along the chromosome is highly significant (*r_s_* = −0.78; 95% CI: −0.94, −0.40; *p*(*r_s_*≥0)<0.001). This result is robust to both the complete removal of *Adh* from the dataset (*r_s_* = −0.67, 95% CI: −0.85, −0.34) or the complete removal of *tim* (*r_s_* = −0.67, 95% CI: −0.87, −0.21). This result is also robust to the underlying sampling variance of the diversity estimates, because bootstrap resampling of the individual bases in the sequence data also demonstrates a significant correlation (median *r_s_* = −0.61; 95% CI: −0.87, −0.17). A similar correlation is observed for standardized heterozygosity (*θ*) using the number of segregating mutations (Supporting Figure S2).

FP was the only sample examined where the frequency of the X-4 fusion is low, so the observed correlation could originate from it having an unusual pattern of sequence diversity along Chromosome 4. Comparison of FP with a previously examined sample from the south [LP from ref. 24] confirms that the finding is not dependent on FP. Silent diversities at *Adh, bib* and *tim* in LP relative to FP are 1.12, 0.95 and 0.94, which exhibit the same rank orders as FP in comparisons with the northern populations. These results demonstrate that sequence diversity in northern relative to southern populations exhibits a distinct pattern along the unfused 4^th^ chromosome. Specifically, the most proximal locus (*Adh*) exhibits higher sequence diversity in populations with a high frequency of fused X chromosomes than in populations with a low frequency of fused X chromosomes, whereas the most distal locus (*tim*) exhibits the inverse relationship. This does not imply that sequence diversity in northern populations is significantly higher than the southern population at *Adh*, or *vice versa* for *tim*, nor does it imply that absolute diversity at proximal loci is greater than distal loci in northern populations. Although the observed differences are small, the overall pattern is extremely robust among samples and across loci.

### Recombination Along Chromosome 4

Measures of linkage relationships between the six loci and the centromere were obtained to quantify the relationship between relative sequence diversity and chromosomal position. The recombination frequency between the centromere and each locus on Chromosome 4 in heterokaryotypic females determines the potential for developing associations between alleles and the sex chromosomes. Genotypes were determined for a total of 336 chromosomes transmitted through females having both a fused and an unfused Chromosome 4. Ten markers representing about 75% of the euchromatic arm of Chromosome 4 were found to encompass a total map distance of 104.6 cM. Centromeric arrangement was determined for 182 chromosomes following their transmission through these heterokaryotypic females, thus providing a direct measure of the recombination frequency for the markers relative to the centromere (Table 4). The *fu1* locus at the base of the X chromosome was also examined and no recombinants were observed in the interval between this locus and the centromere, whereas a single recombinant was observed at the base of Chromosome 4 between the centromere and *Adh*. In 154 chromosomes where the arrangement of the centromere was unknown, two additional recombinants between *Adh* and *fu1* were identified. Even though the crossover events could not be positioned relative to the centromere, these recombinants provide further indication of a low rate of reciprocal exchange between the centromere and *Adh*. Recombination frequency relative to the centromere (*R*) at loci toward the distal end of Chromosome 4 approaches 50% (Table 4), and at locus 3054 and beyond (including the sequenced region *tim*) insignificant LOD scores for linkage between the markers and the centromere were obtained; therefore statistically, these loci assorted independently of the centromere in this limited sample of chromosomes.

**Table 4:**
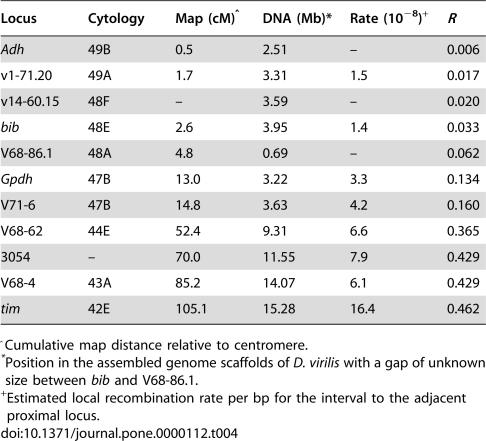
Distribution of loci along Chromosome 4 and estimates of recombination with the centromere (*R*).

Locus	Cytology	Map (cM) ^	DNA (Mb)*	Rate (10^−8^)+	*R*
*Adh*	49B	0.5	2.51	–	0.006
v1-71.20	49A	1.7	3.31	1.5	0.017
v14-60.15	48F	–	3.59	–	0.020
*bib*	48E	2.6	3.95	1.4	0.033
V68-86.1	48A	4.8	0.69	–	0.062
*Gpdh*	47B	13.0	3.22	3.3	0.134
V71-6	47B	14.8	3.63	4.2	0.160
V68-62	44E	52.4	9.31	6.6	0.365
3054	–	70.0	11.55	7.9	0.429
V68-4	43A	85.2	14.07	6.1	0.429
*tim*	42E	105.1	15.28	16.4	0.462

^ Cumulative map distance relative to centromere.

* Position in the assembled genome scaffolds of *D. virilis* with a gap of unknown size between *bib* and V68-86.1.

+ Estimated local recombination rate per bp for the interval to the adjacent proximal locus.

Direct measurements of *R* were obtained for five of the six sequenced regions: *Adh*, v1-71.20, *bib, Gpdh*, and *tim* (Table 4). The v14-60.15 region was included in the sequencing of unfused 4^th^ chromosomes but was not included in the linkage analysis due to the absence of a detectable allelic difference between the parental lines. The genome assembly of *D. virilis*, a species that is closely related to *D americana* and has the same linear arrangement as unfused Chromosome 4, provides a framework for estimating the local recombination rate per bp using the inferred distances between markers in the assembled sequence. The four sequenced regions (*Adh*, v17-12.20, v14-60.15 and *bib*) at the base of Chromosome 4 are located in a single scaffold (GenBank Acc. CH940654.1). Recombination rates in the intervals between mapped markers were used to estimate the local recombinant fraction/bp/generation in heterokaryotypic females at 1.45×10^−8^ for this proximal euchromatic region. Interestingly, local recombination rates increase by 3–10 times this level at medial positions along the chromosome (Table 4). The local recombination rate in the region including v14-60.15 and the physical distance were used to obtain an estimate (*R* = 0.020) for the interval between v14-60.15 and the centromere (Table 4). In summary, the six sequenced regions are well separated along Chromosome 4 and span a range of less than 1% to over 45% recombinants with the centromere.

### Relationship Between Sequence Diversity and Y Linkage

Presence of the X-4 fusion causes sex-linked transmission of Chromosome 4, yet the unfused X is also present in all the populations studied here. Therefore, heterokaryotypic females occur at appreciable frequencies in these populations and they provide opportunities for exchange between the different chromosomal arrangements. Realization of these opportunities depends on linkage relationships with the centromere (Table 4). For example, assuming Hardy-Weinberg and given a frequency of 97% fused X chromosomes for the IR/SB population, 5.8% of females are heterokaryotypes. With a recombination rate of 0.6% between the centromere and *Adh*, alleles at this locus are exchanged between fused and unfused chromosomal arrangements approximately once every 2,900 female generations. Both HI and FP experience an exchange rate of about once every 650 female generations for *Adh* alleles due to similar expected frequencies of heterokaryotypic females. The exchange rate of *tim* alleles is about 75-fold higher than *Adh* in all populations. In the presence of the X-4 fusion, these linkage relationships along Chromosome 4 reflect the potential for establishing disequilibria between alleles on the unfused arrangement and the Y.

**Figure 3:**
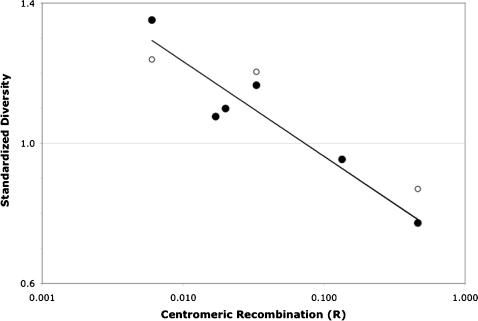
Relationship between mean standardized diversity in northern populations and recombination with the centromere. Filled circles represent mean standardized diversity within the three northern samples (mean HI, IR, SB/FP) plotted in relation to estimated linkage with the centromere on a *log* scale. A significant (*p*<0.01) linear relationship is described by the plotted line, *y* = 0.692−0.271*x*, fitted on the *log* transformed recombinant fraction (*R*). For comparison, unfilled circles are plotted for mean diversity within the three northern samples standardized using a different southern sample [LP from ref. 24].

Sequence diversity was quantified in relation to the gradient of Y linkage that exists along the unfused arrangement of Chromosome 4. A single measure of relative diversity was obtained for each sequenced region by dividing the mean of the pairwise diversities within northern samples (HI, IR, SB) by pairwise diversity within FP. The relationship between relative sequence diversity and the *log* of the recombinant fraction (*R*) for each locus was quantified by linear regression. Consistent with the nonparametric analyses based on chromosomal positions, a significant relationship (*r* ^2^ = 0.89; 95% CI: 0.98, 0.76; *p*<0.01) is observed between standardized sequence diversity and the rate of meiotic exchange between unfused and fused chromosomes (Fig. 3). Based on the regression line [*y* = 0.692−0.271(*log R*)], sequence diversity in northern populations is inflated relative to FP in regions experiencing less than 7.3% recombination with the centromere and reduced relative to FP beyond this point. Although only three data points are available, a similar pattern is apparent along the chromosome when a different southern sample [LP from ref. 24] is used to standardize mean diversity within northern samples (Fig. 3). Because sex-linked transmission of Chromosome 4 is infrequent in the southern populations and frequent in the northern populations, this analysis demonstrates that standardized sequence diversity is highest at loci experiencing the strongest Y linkage and lowest at loci subject to frequent exchange between Y-linked and X-linked contexts.

The apparent lower diversity at *Gpdh* and *tim* for the northern population relative to the southern population may be unrelated to the rate of exchange between neo-Y and neo-X. A previous result indicates that the observed ratio at these medial loci may reflect relative diversities maintained throughout the genome. The one rigorous analysis of sequence diversity in southern and northern populations of *D. americana* for a gene region (*transformer*) located in a non-rearranged chromosomal region indicated a lower level of diversity (relative *π* ∼0.95) in the northern population [36]. If this observation reflects a general pattern throughout the genome, where southern populations maintain higher levels of sequence diversity than northern populations, the inverse relationship at the four proximal loci on Chromosome 4 is even more unusual.

### Propensity for Sexual Antagonism

Hitchhiking with Y-linked sexually antagonistic alleles was investigated as a potential cause of inflated sequence diversity in regions experiencing strong Y linkage. Sexually antagonistic alleles that are advantageous to males but detrimental to females establish polymorphic equilibria when tightly, but not completely, linked with a male-determining locus [26]. Transient Y linkage occurs in *D. americana* due to males with the X-4 fusion transmitting an intact unfused 4^th^ chromosome to their sons. Breakdown of linkage disequilibrium between an antagonistic locus on Chromosome 4 and the Y occurs at two levels: 1) the presence of males with unfused X chromosomes, and 2) the exchange between unfused and fused chromosomes. Thus, it is unclear how polymorphism for the X-4 would affect the evolutionary dynamics of antagonistic alleles along the unfused arrangement.

**Figure 4:**
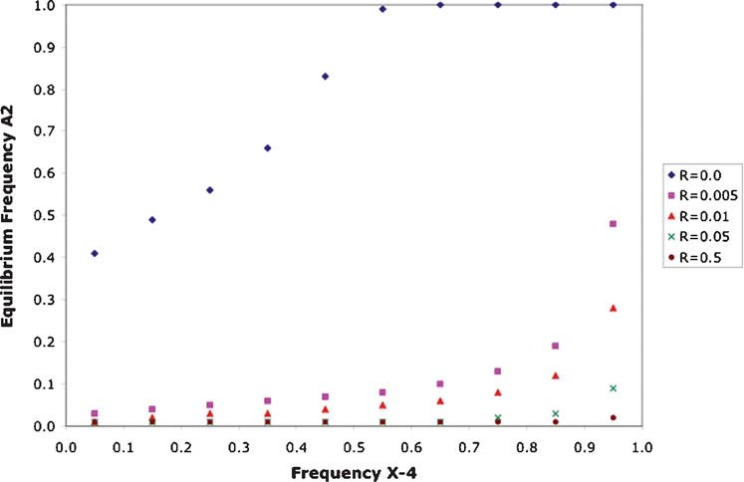
Sexually antagonistic alleles on Chromosome 4. Equilibrium frequencies of a sexually antagonistic allele (*A2*) on the unfused arrangement are plotted for populations segregating the X-4 fusion arrangement. Loci representing a variety of different linkage relationships (*R*) with the centromere are illustrated. Frequency of the X-4 fusion was held constant in the simulations. Results are shown for a completely dominant antagonistic allele with a selection coefficient of 0.001 and fitness effects of opposite sign between males and females. The antagonistic allele increases male fitness and decreases female fitness.

Simulations were conducted to explore the dynamics of a male-benefit/female-detriment allele arising on Chromosome 4 in *D. americana*. Frequency of the X-4 fusion was held constant under the assumption that selective forces maintaining the latitudinal cline are stronger than selection on antagonistic alleles. Relaxing this assumption results in fixation of the X-4 fusion under certain parameters (data not shown). For a dominant allele with a selective advantage in males of 0.001 and an equal disadvantage in females, Figure 4 illustrates the potential to accumulate an antagonistic allele on the unfused arrangement of Chromosome 4 with tight, but incomplete, linkage with the centromere. In populations with greater than 85% of the X chromosomes fused with Chromosome 4 (pertaining to HI, IR & SB), a sexually antagonistic allele increases in frequency and establishes a polymorphic equilibrium when experiencing less than 5% recombination with the centromere (pertaining to *Adh*, v1-71.20, v14-60.15, and *bib*). With the X-4 fusion at a frequency of 95% and a recombination rate with the centromere of 0.5% (similar to the estimates of these parameters for *Adh* in IR and SB), this type of antagonistic allele would be present on approximately half of the unfused 4^th^ chromosomes and would have the greatest potential for increasing flanking neutral variation through hitchhiking. Linkage disequilibrium between the antagonistic allele and the alternative centromeric arrangements is 0.073 (D′ = 0.46), so 12% of fused 4^th^ chromosomes also carry the allele. In contrast, antagonistic alleles experiencing complete linkage with the centromere increase to fixation on the unfused arrangement in populations having a majority of the X chromosomes fused with Chromosome 4 (Fig. 4). These simulations demonstrate that the segregation pattern of Chromosome 4 in populations with both fused and unfused arrangements provides ample opportunity to establish a gradient for accumulating antagonistic variation. This gradient depends on linkage with the centromere and is strongest in populations having the highest frequencies of the X-4 fusion.

## Discussion

A positive relationship between the level of nucleotide diversity and the rate of recombination is a feature of most eukaryotic genomes. Background selection and/or selective sweep processes have received considerable attention as being the underlying cause of this pattern [7], [8], [37], [Bibr pone.0000112-Stephan1] [Bibr pone.0000112-Baudry1] [XPATH ERROR: unknown variable "next".]. While previous studies have generally contrasted genomic regions experiencing chronically low levels of recombination with regions of normal recombination, this study utilized an approach where the same gene regions were compared among populations with different predicted recombination rates. The unfused arrangement of Chromosome 4 is sheltered from crossing over in populations of *D. americana* with a high frequency of the X-4 fusion arrangement, yet the resultant chromosome-wide reduction in recombination rate does not detectably influence observed levels of nucleotide diversity. These results are evaluated in relation to expectations of the background selection and selective sweep models. Furthermore, the observed pattern of increased diversity in the proximal region of Chromosome 4 is considered in relation to evolutionary forces potentially affecting the evolution of sex chromosomes.

Background selection reduces nucleotide diversity in genomic regions with a low rate of recombination as a byproduct of hitchhiking with deleterious mutations that are eliminated from the population. This process could generate a negative correlation between nucleotide diversity on the unfused arrangement of Chromosome 4 and the frequency of the X-4 fusion, but the pattern was not observed. Two factors potentially impede the establishment of different levels of nucleotide diversity as a consequence of background selection. The high level of exchange observed between unfused and fused arrangements of Chromosome 4 may eliminate the effects of background selection. This assertion is supported by the lack of correspondence between predicted per generation reductions in recombination and sequence-based measures of recombination, which indicates that the presence of the X-4 fusion has not detectably affected long-term recombination rates (Supporting Table S1). Gene flow could also homogenize sequence variation across populations [41]. Populations examined in this study were selected to represent a high degree of connectivity with the potential for gene flow, and the observed lack of sequence differentiation is consistent with this potential being realized. Other studies also indicate a high rate of gene flow among populations of *D. americana* [18], [42]. Given frequent recombination and a high rate of gene flow, the failure to detect reduced diversity among these populations as a consequence of background selection is not surprising.

Selective sweeps limited to the unfused arrangement are expected to reduce nucleotide variation more severely in northern relative to southern populations of *D. americana*. This pattern would arise as a consequence of wider windows of reduced neutral variation associated with individual fixation events [7], [43]. Evidence of widespread effects associated with fixation of alleles was not revealed by the contrast among populations. Homogeneity in levels and patterns of nucleotide diversity could result from high rates of gene flow; however, gene flow does not preclude the possibility of local selective sweeps [44], [45]. High rates of gene flow occur among populations of *D. melanogaster*, yet hitchhiking events associated with geographically localized sweeps are evident in its genome [34], [46]. Geographically localized variants also exist in *D. americana*; the X-4 fusion arrangement exhibits a strong latitudinal cline [18], nucleotide variants at the base of the X chromosome are associated with this cline [47], and overlapping paracentric inversions with associated nucleotide variants are limited to the fused arrangement of Chromosome 4 [24]. Even with the high rate of gene flow that apparently exists, these variants indicate the potential for geographically localized adaptive evolution, and yet, a reduction in nucleotide diversity on the unfused arrangement of Chromosome 4 due to selective sweeps is not apparent.

While both background selection and selective sweeps are expected to reduce sequence diversity more severely in the proximal region of Chromosome 4 in northern relative to southern populations, this study revealed the exact opposite pattern. Overall, this analysis identified a robust relationship between nucleotide diversity and Y linkage where loci with the greatest potential for establishing allelic associations with the Y chromosome exhibit the highest standardized levels of nucleotide diversity. Hitchhiking with overdominant alleles is known to increase nucleotide diversity [48]. To account for the observed pattern under such a scenario, however, overdominant alleles must arise as a consequence of Y linkage. Simulations demonstrated the propensity for maintaining an intermediate-frequency sexually antagonistic allele within about 5 cM of the centromere of Chromosome 4. Loci exhibiting polymorphism for such an allele would exhibit overdominant evolutionary dynamics, thus they have the potential to increase neutral variation in flanking regions. Nucleotide diversity along Chromosome 4 may, therefore, be shaped by hitchhiking with sexually antagonistic variation arising as a consequence of Y linkage in the presence of the X-4 centromeric fusion. Other features of the sequence data, namely haplotype structure and frequency spectra, do not exhibit signs of hitchhiking, but it is unclear how strong these effects would be under the ubiquitous antagonism that would be necessary to generate the observed linear relationship between relative diversity and recombination rate with the centromere [49].

The focus of this study was identifying patterns of nucleotide diversity on the unfused arrangement of Chromosome 4. Distinct patterns of nucleotide diversity in relation to the frequency of the X-4 fusion are consistent with Y-linked sexual antagonism shaping variation along this chromosome. The X-4 fusion arrangement should also be prone to X-linked sexual antagonism [50], [51]. Nucleotide diversity for X-linked alleles of *Adh* were previously demonstrated as being higher than Y-linked alleles [23], and although identification of a cause of the asymmetry between X- and Y-linked alleles is confounded by the presence of a nearby chromosomal inversion limited to the fused arrangement [24], nucleotide diversity near the centromere of both arrangements of Chromosome 4 is potentially influenced by antagonistic selective forces.

Chromosome 4 of *D. americana* embodies the dynamics of an emerging pair of sex chromosomes, with the caveat that recombination between the pair occurs in females. Sexual antagonism is widely considered to be the driving force behind the suppression of recombination between emerging sex chromosomes, even though limited empirical evidence supports this inference [17]. Laboratory populations of *D. melanogaster* evolve antagonistic variation very rapidly under experimentally manipulated sex linkage [52], [53], and they have been shown to harbor a large amount of sexually antagonistic variation on the X [51]. Antagonism at the base of Chromosome 4 of *D. americana* would favor a reduction in the rate of recombination between fused and unfused arrangements. Two overlapping paracentric inversions are limited to the fused arrangement of Chromosome 4 and these inversions do suppress recombination with the unfused arrangement [24]. For example, *Adh* and *bib* were shown here to have 0.6% and 3.3% recombinants respectively with the centromere, but none were observed among a similar number of progeny when the X-4 fusion had the inverted arrangement. Both of these genes are located in the interval between the centromere and proximal inversion breakpoint. The inverted region encompasses a large region of the X-4 fusion and the outermost breakpoints are flanked by *bib* and v68-4 (see Figure 1). Suppression of recombination is observed throughout the entire inverted region; inversion heterozygotes produced only 6.9% recombinants between the centromere and *tim* in the previous study compared to the 46.2% observed for the homosequential chromosomes examined here. Identification of a pattern of nucleotide variation consistent with antagonism and the appearance of a recombination suppressor provides two independent indicators that this form of intra-locus sexual conflict is shaping the emerging pair of sex chromosomes in *D. americana*.

Complete Y linkage facilitates the sex-specific fixation of male-benefit/female-detriment alleles by limiting the allele to males and resolving intra-locus conflict. On the unfused arrangement of Chromosome 4, this can occur through complete linkage with the centromere or presence in an isolated population with 100% fused X chromosomes. Neither of these scenarios is likely for the loci and populations analyzed here, and the high level of sequence diversity in the proximal region of unfused Chromosome 4 is inconsistent with recent fixation of any alleles, antagonistic or otherwise. This observation contrasts with the reduced level of variability observed for neo-Y chromosomes of *D. miranda* [54], [55], [56]. Low nucleotide variation for the newly Y-linked region of its genome has been attributed to selective sweeps [57]. Distinct patterns of nucleotide diversity for the neo-Y chromosome of these two Drosophila species are expected, since the different chromosomal rearrangements generating sex linkage bring about distinct dynamics under sexual antagonism [53]. An autosomal arm fused with the ancestral Y to form the neo-Y in *D. miranda*, and thus since its origin, this rearranged chromosome has been completely Y linked. This rearrangement promotes fixation of male-benefit alleles regardless of their fitness effects when expressed in females (female expression is hypothetical, since the alleles are never transmitted to females). Sweeps of male-benefit alleles would fix neutral variation across the entire neo-Y chromosome [58]. This prediction also applies to the Y-limited region of the newly evolved sex chromosome pair, which also exhibits low nucleotide diversity, in the plant genus *Silene* [14], [59]. Female fitness matters for alleles on the transient neo-Y of *D. americana*, and therefore, polymorphism is the expected outcome of intra-locus sexual antagonism. Sequence analysis of the pseudoautosomal regions of the *Silene* sex chromosomes would potentially reveal a pattern similar to the one reported here, especially given the evidence these regions contain male-limited QTL influencing sexually antagonistic floral traits [60].

If sexual antagonism shapes patterns of nucleotide diversity on Chromosome 4 of *D. americana*, this could be an indicator of the long-term consequences for this chromosomal pair. Sexual antagonism may interfere with local adaptation on the unfused arrangement of Chromosome 4, since there is currently no evidence of local adaptation influencing patterns of nucleotide diversity. Degeneration of gene function is a key feature of evolving Y chromosomes, and the interference generated by sexually antagonistic alleles could contribute to this process [4], [5]. Hitchhiking of deleterious mutations with sexually antagonistic alleles is a form of interference previously proposed to facilitate Y-chromosome degeneration [58]. Intriguingly, the pattern revealed here has emerged prior to any apparent gene loss from the neo-Y chromosome [61]. Direct demonstration of sexually antagonistic genetic variation at the base of Chromosome 4 in northern populations of *D. americana* would further elucidate its influence on this emerging pair of sex chromosomes.

## Materials and Methods

### Populations Examined

Sequences were obtained as haplotypes from unfused 4^th^ chromosomes derived from four samples of *D. americana*. The four samples were collected at separate localities in the central United States, and they represent latitudinally distributed populations differing in frequency of the X-4 fusion. Samples of FP and HI were collected in 1999 and described previously [18]. FP was collected near the White River in southeastern Arkansas, and HI was collected near the Missouri River west of St. Louis, Missouri. SB was sampled in September 2002 from the Saulsbury Bridge Recreation Area near the Cedar River, Muscatine County, Iowa (GPS: 41.495°N, 91.161°W). A total of 24 independent X chromosomes were examined using a modification of previously described methods [18], and all X chromosome were identified as being fused with the 4^th^ chromosome. IR was sampled in July 2004 from the Hawkeye Wildlife Area on the Iowa River, Johnson County, Iowa (GPS: 41.779°N, 91.715°W). A total of 117 X chromosomes were examined, and 114 were fused with Chromosome 4.

In addition to the observed data for each sample, the proportion of X chromosomes fused with Chromosome 4 and 95% CI was estimated for each population from a logistic regression model based on geographic coordinates. A total of 641 X chromosomes representing 13 distinct geographic localities in the central United States were included in the model (McAllister et al. unpublished data). Details of the model and the samples upon which it is based will be published separately.

Frequency of the X-4 fusion impacts the occurrence of meiotic recombination involving unfused 4^th^ chromosomes. This arises from the restriction of unfused 4^th^ chromosomes in males, which lack normal meiotic crossing over. Given *p*, the proportion of X chromosomes fused with the 4^th^ chromosome, the proportion of unfused 4^th^ chromosomes in females each generation is 2(1−p)/4−3p. Assuming this proportion is 1/2 in the absence of the X-4 fusion, the expression 4(1−p)/4−3p estimates the reduction in the recombination rate (*r*) between two loci on the unfused 4 relative to the autosomal condition.

### Sequence Data

Sequence diversity was measured at six positions distributed along the 4^th^ chromosome in these four samples. Characteristics of the sequenced regions are provided in Table S2 (Supporting Data). Single F1 hybrids containing an unfused Chromosome 4 of *D. americana* and its homolog from *D. virilis* were used as template for amplification of each locus using *D. americana*-specific primers. These male hybrids were obtained from crosses of single wild-caught males, or a single male from an iso-female line, to the V46 strain of *D. virilis*. Presence of an unfused 4^th^ chromosome is ensured by only sequencing from an F1 son of a *D. americana* male (*i.e.*, the male can have either a fused or unfused X, but the son will always inherit a single unfused 4). Amplified PCR product was sequenced directly using an ABI 3100 or 3730. Traces from forward and reverse reads were trimmed, aligned, and manually edited using Sequencher (Gene Codes, Ann Arbor, MI). An outgroup sequence of each region was obtained from the whole genome assembly of *D. virilis* (caf1, Agencourt Biosciences, Beverly, MA). The set of sequences was aligned manually, and boundaries between coding and noncoding regions were annotated according to orthologous gene sequences in *D. melanogaster*, except for the annotation of the coding region in v14-60.15 (see below).

Primers used to amplify and sequence regions of the *Alcohol dehydrogenase* (*Adh*), *big brain* (*bib*) and *timeless* (*tim*) genes have been described previously [23], [24]. Reported cytological positions of *Adh* and *bib* are near the centromere of Chromosome 4 respectively at positions 49B and 48E on the Gubenko and Evgen'ev [62] photographic map of *D. virilis*, whereas *tim* is located at position 42E toward the distal end of the chromosome [18], [63], [61]. Primers specific for the amplification of a region of *Glycerol 3 phosphate dehydrogenase* (*Gpdh*) were designed based on the sequences of Tominaga and Narise [64]. Primer sequences for amplification and initial sequencing are: 5′ tttgccagggtccagttg, and 5′ cttggatatacaaagtggtacgt. Full sequences of both strands were obtained using internal sequencing primers. This gene was localized to cytological band 47B of *D. virilis* by *in situ* hybridization following standard procedures [61].

Sequences were also determined for two regions in the interval between *Adh* and *bib*. Regions were initially targeted in this interval by obtaining sequence from subclones generated from P1 clones v1-71 mapped to subdivision 49A and v14-60 mapped to subdivision 48F of the *D. virilis* genome [65], [66]. Cells' containing the P1 clones of *D. virilis* genomic DNA were generously provided by Jorge Vieira (Universidade do Porto). Primers developed from the *D. virilis* sequence were used to amplify and sequence the corresponding region in a set of inbred lines of *D. americana*, therefore, enabling the development of species-specific primers for amplification and sequencing of *D. americana* alleles from hybrid flies. Primer sequences for amplification and sequencing of v1-71.20 are: 5′ ttgtagccaccacctcca, and 5′ aYactgtgggcggttattc. Primer sequences for v14-60.15 are: 5′ ttgcggctcggagaacag, and 5′ catcgaggctcggatctt. Sequence of the v1-71.20 region corresponds to a portion of CG17549 of *D. melanogaster*, and the v14-60.15 region corresponds to a portion of CG5682. Exon/intron boundaries of the sequenced regions were determined by MultiPipMaker alignments [67] with genomic and cDNA sequences of *D. melanogaster*. Two intron splice sites surrounding a central exon of CG17549 appear to be conserved between the *virilis* species group and *D. melanogaster*; however, an intron annotated in CG5682 of *D. melanogaster* appears as an insertion relative to the sequences of this gene in *D. americana* and *D. virilis*.

### Sequence Analyses

The primary goal of the study was to test for effects on the unfused arrangement of Chromosome 4 arising as a consequence of the X-4 fusion by examining patterns across populations and among loci. The total dataset consists of approximately 182 kb of sequence distributed evenly among four samples each with ten independently sampled chromosomes. Sequences were derived from six regions distributed along Chromosome 4, and the individual regions range in size from 502 bp to 1,141 bp encompassing a total aligned length of 4,566 bp (details in Supporting Table S2). Analyses of the sequence data were performed using DnaSP v4.10 [68].

Statistics based on the distribution of variation within samples did not exhibit detectable patterns across populations or loci, and therefore, only Tajima's *D* [30] calculated from the total number of mutations is reported. Pairwise and overall population differentiation was estimated with the *K_st_* statistic [69]. Significance of observed values of this statistic relative to the expectation of zero was assessed by permutation tests. Estimates of the recombination parameter (*4Nr*) were obtained directly from the sequences [70], and the results are presented in Supporting Table S1.

Reported analyses of sequence diversity are based on measures of pairwise differences [71], but the same general results were obtained for diversity estimates based on numbers of segregating sites, numbers of mutations, or diversity relative to divergence (using *D. virilis* as the outgroup). Due to heterogeneity in silent (synonymous and noncoding) diversity among genes, total pairwise diversity in the three northern samples was divided by pairwise diversity within the southern sample (FP) to standardize among genes and examine patterns along the chromosome. Tests for homogeneity in pairwise diversity (and other variables) and correlations were performed using SAS ver. 9.1.3 (Cary, NC). Differences in means were evaluated on rank scores by invoking Cochran-Mantel-Haenszel statistics and testing for homogeneity (Friedman 2-way ANOVA) in means [72]. Correlations were measured using PROC CORR, and confidence intervals were obtained with the JACKBOOT macro as percentage estimates of the distributions from 10,000 bootstrap samples of the data [73].

A customized routine for bootstrap resampling of the sequence data was compiled using Absoft Pro Fortran 8.0 (Rochester Hills, MI). Individual bases in the sequence data were sampled with replacement, and the Spearman Rank Correlation (*r_s_*) was obtained from the standardized diversities (divided by FP) of the three northern samples (HI, IR, SB) against chromosomal position. The median *r_s_* from the 10,000 replicates reflects the correlation based on the median pairwise diversity and the confidence interval obtained from the distribution of the statistic for the resampled data reflects the underlying variance in the estimate of pairwise diversity. The proportion of replicates where *r_s_* in the resampled data was equal to or greater than zero indicates the probability of this outcome given these sequence data.

### Recombination Estimates

The recombination rate in heterokaryotypic females with alternative centromeric arrangements of Chromosome 4 was assayed directly from laboratory crosses. Reciprocal crosses were established between inbred lines G96.23 and ML97.5. The former line is fixed for an X-4 centromeric fusion and the later line is fixed for an unfused X chromosome. Both lines have the standard linear arrangement of Chromosome 4 for the *virilis* species group, which was checked following standard procedures for preparation of polytene chromosomes from the salivary glands of F1 larvae [74]. The same crossing design applied by McAllister [24] was used to generate single flies with X and 4^th^ chromosomes transmitted through a single female meiosis. Centromeric arrangement was inferred from segregation of the eye-color mutation at the *cardinal* (*cd*) locus, which is on Chromosome 4. The *cd* locus segregates as an autosomal recessive when the X and 4^th^ are unfused and as a sex-linked diploid recessive (*i.e.*, pseudoautosomal locus in complete linkage with gender) when the X and 4^th^ are fused. Additional flies were obtained with X and 4^th^ chromosomes transmitted through the same design, but where the centromeric arrangement was not inferred due to the number of progeny being insufficient for identifying a statistical association (*p*>0.05) between the *cd* locus and gender. DNA was extracted from flies using the DNeasy 96 tissue kit (Qiagen).

Genotypes of recombinant chromosomes were determined for ten loci on Chromosome 4 and one locus on the X chromosome. Assays were either based on discrimination of parental alleles using restriction site differences in PCR products or size differences at microsatellite loci (Supporting Table S3). The RFLP loci were screened on 1.5% agarose gels. Microsatellite loci were amplified with one primer having 5′ conjugated FAM or HEX and were screened in multiplex runs on an ABI 3100 using Genescan software to analyze the trace output (Applied Biosystems, Foster City, CA). The genotype data were analyzed with Map Manager QTb29ppc [75] to estimate intervals between loci using the Kosambi mapping function and to obtain the recombinant fraction (*R*) relative to the centromere.

A polymorphic marker discriminating the lines was not present in the v14-60.15 sequenced region, so the rate of recombination with the centromere was not directly measured, but inferred from the local recombination rate. The physical distance between the markers was estimated based on the genome assembly of the closely related species *Drosophila virilis* (caf1, Agencourt Biosciences, Beverly, MA). An approximately 100-bp window including each marker was queried against the assembled genome sequence using *blastn* [76] to identify the position of the marker locus. The distance between markers was estimated from the size of the intervening sequence and estimated gaps. Two sets of markers were identified in separate scaffolds, and due to the presence of a gap of unknown size between these scaffolds, the interval between adjacent markers (*bib* and v68-86.1) separated by this gap was not estimated.

### Computer Simulations

Evolutionary dynamics of sexually antagonistic alleles on Chromosome 4 of *D. americana* were simulated according to a modified model of Rice [26] using code compiled with Absoft Pro Fortran 8.0 (Rochester Hills, MI). The frequency of the X-4 fusion was maintained at a constant value, whereas the frequency of an antagonistic allele (A2) changed deterministically from an initial value of 0.01 according to specified selection coefficients. Equilibrium frequencies of a completely dominant (*h* = 1.0) A2 allele with equal selection coefficients (0.001) of opposite sign for males and females were obtained for a range of frequencies of the X-4 fusion. A relatively low value of selection was used under the assumption that the selective force maintaining the cline for the X-4 fusion is stronger than selection on the antagonistic allele, because in simulations where the frequency of the fusion is free to change, the antagonistic allele can drive the fusion to fixation (data not shown). Simulations were initiated with the chromosomal arrangements and the antagonistic alleles in linkage equilibrium and were continued until there was no change in the frequency of the A2 allele.

## Supporting Information

Table S1 Sequence-based estimates of the recombination parameter
(0.03 MB DOC)

Table S2 Descriptions of the sequenced regions
(0.02 MB DOC)

Table S3 Assay methods for loci used to measure linkage relationships
(0.03 MB DOC)

Figure S1 Pairwise sequence diversity in *D. americana* relative to fixed differences in comparison with *D. virilis*. Numbers of fixed differences at each locus were relative to the entire set of 40 sequences from *D. americana*. Loci are arranged according to relative position along Chromosome 4 with the centromere toward the left and telomere toward the right.
(0.05 MB TIF)

Figure S2 Standardized estimate of heterozygosity (θ) measured from the number of mutations. Observed θ in each northern sample is standardized using θ for the southern FP sample. A significant Spearman rank correlation is obtained relative to position on Chromosome 4 (r_s_ = −0.78, 95% CI: −0.91, −0.57).
(0.04 MB TIF)
