# Supplementary material for: Increased Nucleotide Diversity with Transient Y Linkage in Drosophila americana
Source: PLoS One. 2006 Dec 27;1(1):e112. doi: 10.1371/journal.pone.0000112 (PMC1762432; doi:10.1371/journal.pone.0000112)
Supplement: Table S1 — Sequence-based estimates of the recombination parameter (0.03 MB DOC) [file pone.0000112.s001.doc]

Supplementary Table S1. Sequence-based estimates of the recombination parameter

|  | FP | HI | IR | SB | Total |
| --- | --- | --- | --- | --- | --- |
| Adh *R_H_*  *r* per bp | 747  0.89 | >10000  - | 521  0.62 | 293  0.35 | 786  0.94 |
| v1-71.20  *R_H_*  *r* per bp | 596  1.13 | 300  0.57 | >10000  - | 32.9  0.06 | 130  0.25 |
| v14-60.15  *R_H_*  *r* per bp | 319  0.54 | 144  0.24 | 90.5  0.15 | 35.1  0.06 | 95.3  0.16 |
| *bib*  *R_H_*  *r* per bp | 106  0.11 | 436  0.47 | 153  0.16 | 111  0.12 | 123  0.13 |
| Gpdh *R_H_*  *r* per bp | 2304  2.02 | 4896  4.29 | 442  0.39 | 326  0.29 | 621  0.55 |
| *tim*  *R_H_*  *r* per bp | >10000  - | 134  0.27 | >10000  - | 46.8  0.09 | 156  0.31 |

*R_H_* corresponds to the Hudson [[[1]](#endnote-2)] estimator of *4Nr* for the entire region, and when informative, the corresponding rate between adjacent sites is presented.

1. [?] Hudson RR (1987) Estimating the recombination parameter of a finite population model without selection. Genet Res 50: 245-250. [↑](#endnote-ref-2)
