# Supplementary material for: Increased Nucleotide Diversity with Transient Y Linkage in Drosophila americana
Source: PLoS One. 2006 Dec 27;1(1):e112. doi: 10.1371/journal.pone.0000112 (PMC1762432; doi:10.1371/journal.pone.0000112)
Supplement: Table S2 — Descriptions of the sequenced regions (0.02 MB DOC) [file pone.0000112.s002.doc]

Supplementary Table S2. Descriptions of the sequenced regions

|  | Cytology | Characteristics | Amplification Primers | Temp |
| --- | --- | --- | --- | --- |
| Adh | 49B | 846 bp aligned  181 synonymous  124 5’ UTR+intron | 5’ ccgactagaaagcatcac  5’ atttgaatggtttagatatgc | 54˚ |
| v1-71.20  (CG17549) | 49A | 530 bp aligned  47 synonymous  342 intron | 5’ ttgtagccaccacctcca  5’ aYactgtgggcggttattc | 56˚ |
| v14-60.15  (CG5682) | 48F | 594 bp aligned  143 synonymous | 5’ ttgcggctcggagaacag  5’ catcgaggctcggatctt | 58˚ |
| *bib* | 48E | 953 bp aligned  164 synonymous  179 intron | 5’ tacgatttcggacttgcgaa  5’ atgctctctgtacgctgttg | 58˚ |
| Gpdh | 47B | 1141 bp aligned  174 synonymous  361 intron | 5’ tttgccagggtccagttg  5’ gggaatctgcaagtatagt | 61˚ |
| *tim* | 42E | 502 bp aligned  112 synonymous | 5’ gatccgaagagcaccaagag  5’ gtggcatcggtgccctca | 60˚ |
