# Supplementary material for: Increased Nucleotide Diversity with Transient Y Linkage in Drosophila americana
Source: PLoS One. 2006 Dec 27;1(1):e112. doi: 10.1371/journal.pone.0000112 (PMC1762432; doi:10.1371/journal.pone.0000112)
Supplement: Table S3 — Assay methods for loci used to measure linkage relationships (0.03 MB DOC) [file pone.0000112.s003.doc]

Supplementary Table S3. Assay methods for loci used to measure linkage relationships.

| Locus | Cytology | Primers (5’-3’) | Assay | Reference |
| --- | --- | --- | --- | --- |
| *fu1* | 18C | gccgagtgacattgagcag  gctcggcgttctcgggtag | RFLP-*Cla*I | Vieira et al. 2001 [[[1]](#endnote-2)] |
| Adh | 49B | ccgactagaaagcatcac  atttgaatggtttagatatgc | RFLP-*Hha*I | McAllister 2002 [[[2]](#endnote-3)] |
| v1-71.20 | 49A | ttgtagccaccacctcca  ayactgtgggcggttattc | RFLP-*Alu*I | This study |
| *bib* | 48E | tacgatttcggacttgcgaa  ggtgtacagattctggcag | RFLP-*Bbr*PI | McAllister 2002 [3] |
| v68-86.1 | 48A | tcgcatctcaaaatttaattgac  agcagcaaaacgaagcaaat | MS-(TG)_n_ | Schlötterer 2000 [[[3]](#endnote-4)] |
| Gpdh | 47B | gttctattggccgcttatc  tcggcattcttggcagcctc | MS-(CA)_n_ | This study |
| v71-6 | 47B | cgaacagtttagccagaa  acagataaacagttgcacag | MS-(GT)_n_ | Schlötterer 2000 [3] |
| v68-62 | 44E | atgttgcaagctgttgttgc  tcggtgtcattcgtaggttg | MS-(GTT)_n_ | Schlötterer 2000 [3] |
| v3054 | ? | attcaatatctccaggctccc  ttctgtgttatgctgcctgctgaac | MS-(GA)_n_ | This study |
| v68-4 | 43A | aagacgggctgaggatgtgag  aacaataaatgatgtgaaatc | MS-(CA)_n_ | Schlötterer 2000 [3] |
| *tim* | 42E | ttcaatatctccaggctccc  gtttgccgtcaaagtgtagaac | MS-(GA)_n_ | This study |

1. [?] Vieira J, McAllister BF, Charlesworth B (2001) Evidence for selection at the *fused1* locus of *Drosophila americana*. Genetics 158: 279-290. [↑](#endnote-ref-2)
2. [?] McAllister BF (2002) Chromosomal and allelic variation in *Drosophila americana*: selective maintenance of a chromosomal cline. Genome 45: 13-21. [↑](#endnote-ref-3)
3. [?] Schlötterer C (2000) Microsatellite analysis indicates genetic differentiation of the neo-sex chromosomes in *Drosophila americana americana*. Heredity 85: 610-616. [↑](#endnote-ref-4)
