# Supplementary figures and images for: Increased Nucleotide Diversity with Transient Y Linkage in Drosophila americana
Source: PLoS One. 2006 Dec 27;1(1):e112. doi: 10.1371/journal.pone.0000112 (PMC1762432; doi:10.1371/journal.pone.0000112)

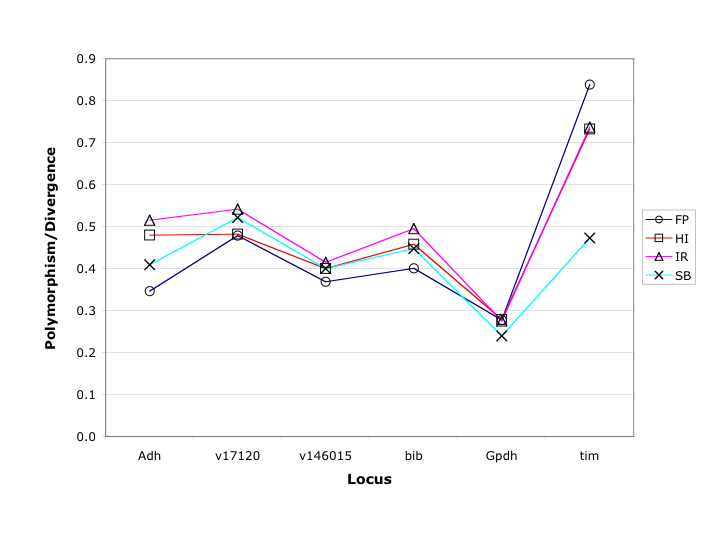

Supplement: Figure S1 — Pairwise sequence diversity in D. americana relative to fixed differences in comparison with D. virilis. Numbers of fixed differences at each locus were relative to the entire set of 40 sequences from D. americana. Loci are arranged according to relative position along Chromosome 4 with the centromere toward the left and telomere toward the right. (0.05 MB TIF) [file pone.0000112.s004.tif]

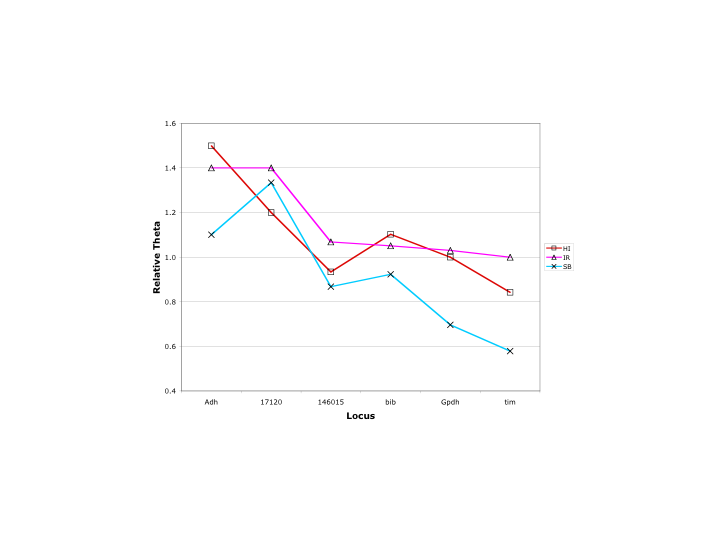

Supplement: Figure S2 — Standardized estimate of heterozygosity (θ) measured from the number of mutations. Observed θ in each northern sample is standardized using θ for the southern FP sample. A significant Spearman rank correlation is obtained relative to position on Chromosome 4 (rs = −0.78, 95% CI: −0.91, −0.57). (0.04 MB TIF) [file pone.0000112.s005.tif]
